# *Helicobacterpylori* Infection—A Risk Factor for Irritable Bowel Syndrome? An Updated Systematic Review and Meta-Analysis

**DOI:** 10.3390/medicina58081035

**Published:** 2022-08-02

**Authors:** Ziyan Wang, Yuhua Liu, Yinglong Peng, Liang Peng

**Affiliations:** 1 Department of Gastroenterology, The First Affiliated Hospital of Guangzhou Medical University, Guangzhou 510120, China; wangzy2018@stu.gzhmu.edu.cn (Z.W.); 2018111110@stu.gzhmu.edu.cn (Y.L.); 2 The First Clinical School, Guangzhou Medical University, Guangzhou 510120, China; 3 School of Medicine, South China University of Technology, Guangzhou 510006, China; pengricardo930@gmail.com

**Keywords:** irritable bowel syndrome, functional gastrointestinal disorders, *Helicobacter pylori*, meta-analysis

## Abstract

Nowadays, the relationship between *Helicobacter pylori* infection (HPI) and irritable bowel syndrome (IBS) remains controversial. Objective: The aim of this study is to investigate the relationship between HPI and IBS through a systematic review and meta-analysis based on the current evidence. Methods: We performed a systematic literature search in electronic databases (PubMed, EMBASE, and the Cochrane library) by computer to identify all reports published before 8 August 2021. The odds ratio (OR) and confidence interval (CI) were calculated to evaluate the association between HPI and IBS. Subgroup analyses were conducted for further assessment and exploration of heterogeneity sources. In addition, we assessed publication bias through funnel plots, Egger’s test, and Begg’s test. Finally, we conducted a sensitivity analysis to evaluate the robustness of the results. Results: Thirteen studies with 13,173 participants were included in the meta-analysis. The pooled OR of the association between HPI and IBS was 1.03 (95% CI [0.80,1.31]; *p* = 0.84). The adjusted OR of the association between HPI and IBS after excluding the studies with confounding factors defined by our team was 1.29 (95% CI [1.03,1.62]; *p* = 0.03). We found a positive association between HPI and IBS-D (diarrhea subtype) (OR: 1.54; 95% CI [1.22,1.95]; *p* = 0.0003). The OR of the relationship between cytotoxin-associated gene A (Cag A) positive HPI and IBS was 4.3 (95% CI [0.51,36.17]; *p* = 0.18). Conclusions: The likelihood of HPI in IBS patients is relatively higher than that of non-IBS participants but not statistically significant, implying that HPI is not significantly associated with IBS, albeit we may underestimate this association. Moreover, we found a positive association between HPI and IBS-D. We also observed an increased likelihood of Cag-A positive HPI in IBS patients than that of non-IBS participants but not statistically significant.

## 1. Introduction

Irritable bowel syndrome (IBS) is a disorder characterized by abdominal discomfort and changes in bowel habits that are unable to be attributed to any other gastrointestinal condition [1,2]. IBS can be divided into four major subtypes including diarrhea type (IBS-D), constipation type (IBS-C), the mixed type of diarrhea and constipation (IBS-M), and the unspecified type (IBS-U) according to the bowel habits or property of the stool. The pooled prevalence rate of IBS is 4.1%, according to the findings of an internet survey conducted in 54,127 individuals from 26 countries [3]. Based on primary care registers, IBS exhibits long-term associations with psychosocial problems, somatic symptoms, and physical diseases of different systems [4]. IBS is the cause of distress, morbidity, and disability, which severely affect the quality of life of individuals and impose a substantial burden on the healthcare system [1,5]. Therefore, to find the appropriate treatment for IBS, it is necessary to identify its risk factors. The pathogenesis of IBS can be explained through various hypotheses. A recent review showed that the gut-brain axis theory might explain the pathogenesis of IBS, with associated mechanisms such as infection and disturbances in gut microbiota, inflammation, bile acid malabsorption, visceral hyperalgesia, mental disorders, alteration of the central processing of afferent stimuli, and gene disorders [6]. In particular, an increasing number of studies have found that microorganisms are involved in the pathogenesis of IBS. For instance, the family Enterobacteriaceae, family Lactobacillaceae, genus Bacteroides, and phylum Proteobacteria have been identified to be related to IBS [7]. These evidence suggested that microbiota was involved in the development of IBS.

*Helicobacter pylori* (*Hp*), a type of gram-negative bacterium that can resist highly acidic conditions, lives on the epithelial surface of the stomach and is highly prevalent worldwide [8,9,10]. *Helicobacter pylori* infection (HPI) has been associated with several intra-gastric and extra-gastric diseases, such as gastroesophageal reflux [11], functional dyspepsia [12], and chronic cholecystitis and cholelithiasis [13]. Several researchers have reported that HPI might promote the development of IBS [14,15,16]. Furthermore, some researchers have also reported that *Hp* eradication therapy can decrease the future risk of IBS in clinical practice [15]. However, other studies concluded that HPI does not induce IBS, and *Hp* eradication therapy is ineffective in treating IBS [16,17]. Thus, there is still a controversial association between HPI and IBS. Although some meta-analyses that pertain to this issue have been previously carried out [18,19,20], these studies did not further examine the impact of participants’ health status on the relationship between HPI and the IBS, the relationship between HPI and the different subtypes of IBS, and the relationship between cytotoxin-associated gene A (Cag A) positive HPI and IBS. Therefore, it is necessary to examine further the relationship between HPI and IBS through a systematic review and meta-analysis of additional published reports and additional analyses.

## 2. Methods

This study followed the Preferred Reporting Items for Systematic Reviews and Meta-Analyses (PRISMA) guidelines [21]. We registered this study in the International Prospective Register of Systematic Reviews (PROSPERO, CRD42021283097).

### 2.1. Eligibility Criteria

Studies with the following criteria were included: (1) study population: hospital-based population or community-based population; (2) case group: IBS patients; (3) control group: non-IBS patients; (4) outcome: HPI rate in IBS patients and non-IBS participants; and (5) study design: observational study. Studies meeting the following criteria were excluded: (1) studies unrelated to humans; (2) abstracts, reviews, editorials, notes, letters, or case reports; (3) studies lacking necessary data or full text; or (4) studies with inappropriate study designs.

### 2.2. Information Sources, Search Strategy, and Selection Process

**Figure 1:**
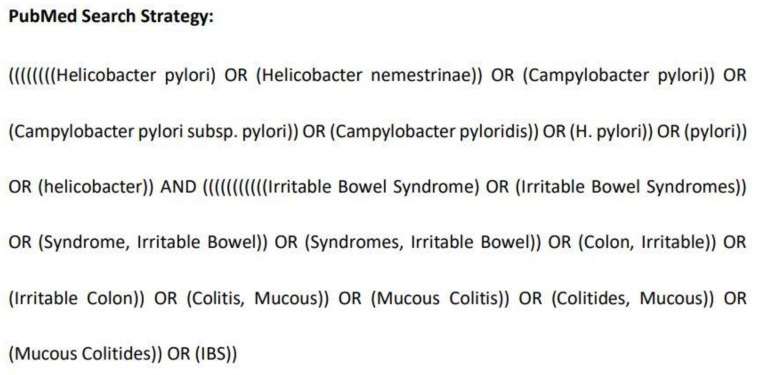
The detailed search strategy used in PubMed.

Public electronic databases, including PubMed, EMBASE, and the Cochrane Library, were independently searched by two reviewers (Z.W. and Y.L.) for studies up to 8 August 2021. One reviewer designed the searches, to which the other authors agreed. The detailed search strategy used in PubMed is presented in Figure 1. The detailed search strategies used in EMBASE and the Cochrane Library are outlined in Supplementary Material S1. Two reviewers (Z.W. and Y.L.) independently conducted the selection process, and any differences were resolved via discussion with another reviewer (L.P.). All records were imported into Endnote X9 (Clarivate Analytics, Philadelphia, USA). Duplicated records were removed by automation tools in Endnote X9 and manually by one reviewer (Z.W.). Two reviewers (Z.W. and Y.L.) reviewed the full texts of the remaining records to select the texts that satisfied the eligibility criteria, and any differences were resolved via consensus.

### 2.3. Data Extraction

Two reviewers (Z.W. and Y.L.) extracted all available data from the included studies into Excel 2019 (Microsoft, Redmond, WA, USA). The following data were extracted: the last name of the first author, publication year, region, study design, studied population, IBS diagnostic criteria, the subtypes of IBS, *Hp* detection method, the virulence factor of *Hp*, age, sex, number of IBS patients, number of *Hp*-positive IBS patients, number of all non-IBS participants, number of *Hp*-positive non-IBS participants, the status of IBS patients, and status of non-IBS participants. Any differences in the extracted data were resolved via discussion with another reviewer (L.P.).

### 2.4. Quality Evaluation

We used the Newcastle–Ottawa Scale (NOS), which is a set of scales with seven or eight items that can be rated up to nine stars, to evaluate the quality of the case-control study and cohort study. The studies were rated from three perspectives: selection, comparability, and exposure (or outcome). There is no universally accepted definition of high-quality studies. Those studies that received more than five stars were considered high-quality studies. A quality evaluation scale with 11 items, designed by the Agency for Healthcare Research and Quality (AHRQ, Rockville, MD, USA), was used to evaluate the quality of the cross-sectional study. Higher scores were given to studies if they met more items (a total of 11 items). Higher scores indicated the higher quality of the studies. Two reviewers (Z.W. and Y.L.) performed the quality evaluation independently and resolved any differences through discussion with another reviewer (L.P.).

### 2.5. Statistical Analysis

The overall odds ratio (OR) of all studies, ORs of each study, 95% confidence intervals (CIs), and P values were calculated. Cochran’s Q test and I^2^ statistics were calculated to assess the existence and magnitude of heterogeneity. In the Q test, *p* < 0.1 indicated the presence of statistical significance [22,23,24]. An I^2^ value over 50% was considered to be moderate to high [22], which we defined as remarkable. When *p* < 0.1 or I^2^ > 50%, the random-effects (RE) model was employed for calculation. Otherwise, we used the fixed-effects (FE) model. Moreover, we performed subgroup analyses to investigate the link between HPI and IBS further and determine the sources of heterogeneity considering five factors: (1) region; (2) study population; (3) IBS diagnostic criteria; (4) *Hp* detection method; and (5) study design. If the I^2^ values of all the subgroups under the target factor were less than the pooled I^2^ of the factor, this factor was considered a possible source of heterogeneity. To further investigate the relationship between HPI and IBS, we excluded studies with confounding factors defined by our team, and the adjusted OR was calculated to improve the accuracy of the results. The criteria for studies that contained confounding factors were as follows: (1) studies that reported that participants suffered from any gastrointestinal disorder other than IBS and (2) studies in children. In addition, we examined the relationship between HPI and different subtypes of IBS. Moreover, the OR of the association between Cag A-positive HPI and IBS was calculated. We assessed publication bias through funnel plots, Egger’s test [25], and Begg’s test [26], and *p* < 0.1 implied significant publication bias. In addition, the robustness of the results was assessed by sensitivity analysis, excluding each study consecutively. All P values are two-tailed. The results were treated as statistically significant if *p* < 0.05 (except for Cochran’s Q test, Egger’s test, and Begg’s test). Review Manager 5.4 (Cochrane Collaboration, Copenhagen, Denmark) and Stata 15 (Stata Corp, College Station, TX, USA) were used for all statistical analyses.

## 3. Results

### 3.1. Study Selection

**Figure 2:**
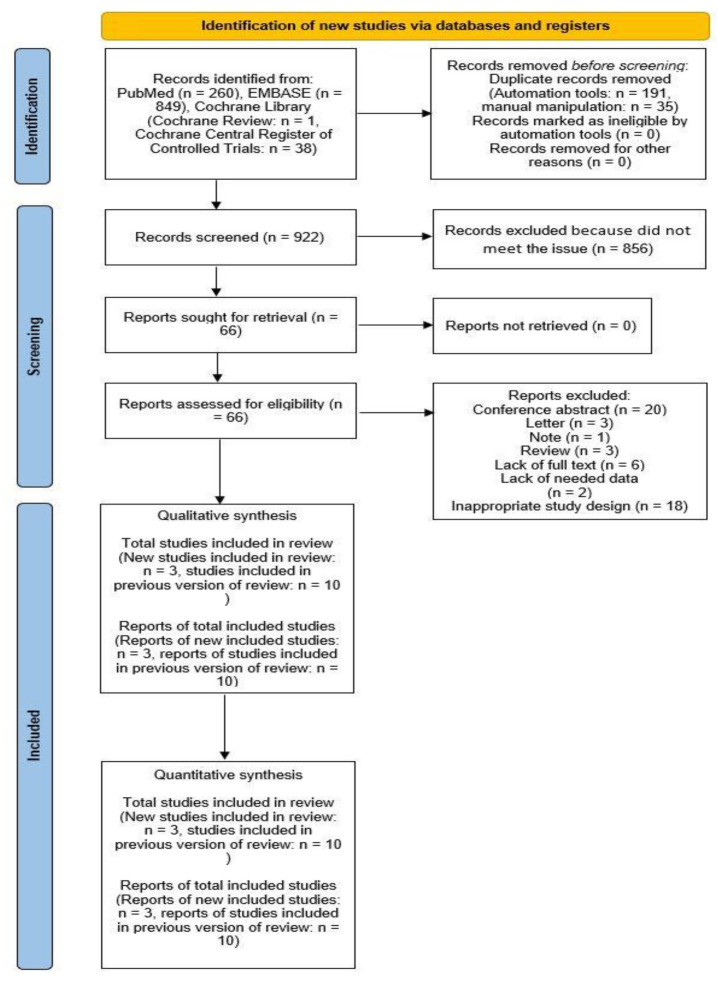
PRISMA flow of study selection. Previous version of review, the previously published meta-analysis or systematic reviews regarding the similar topic. New studies, the studies not included in previously published meta-analysis or systematic reviews regarding the similar topic.

In total, 1148 records were extracted from the public databases, including PubMed (n = 260), EMBASE (n = 849), and the Cochrane Library (Cochrane Central Register of Controlled Trials: n = 38, Cochrane Review: n = 1). Moreover, 226 duplicate records were removed (automation tools: n = 191, manual removal: n = 35). Of the remaining 922 records, 856 were removed because they did not meet the criteria. The remaining 66 records were retrieved for full-text review. These 66 reports were then assessed for eligibility by reading the full texts. Accordingly, 52 of the 66 reports were excluded for the following reasons: conference abstract (n = 20), letter (n = 3), note (n = 1), review (n = 3), lack of full text (n = 6), lack of necessary data (n = 2), and inappropriate study design (n = 18). Thirteen studies were included in the meta-analysis [14,16,27,28,29,30,31,32,33,34,35,36,37]. Notably, three additional studies were included in the meta-analysis [27,28,35], which were not included in the previously published meta-analyses or systematic reviews on similar topics. The study selection process is given in Figure 2.

### 3.2. Study Characteristics

The characteristics of the included studies in the meta-analysis are shown in Table 1. The results of the quality assessment for each study included in the meta-analysis are presented in Supplementary Material S2. The detailed numbers of all patients and participants and the funding information of all included studies are presented in Supplementary Material S3. The studies were published between 1995 and 2019, with six from Asia, five from Europe, one from North America, and one from Africa. The study design of all the included studies was observational, including eight case-control studies and five cross-sectional studies. Moreover, five studies were community-based, while eight studies were hospital-based. Concerning the IBS diagnostic criteria, ten studies adopted the Rome criteria, two studies adopted the Manning criteria, and one study adopted criteria designed by the authors. Regarding the *Hp* detection method, six studies used immunological methods, two studies used rapid urease test (RUT), two studies used histological methods, two studies used comprehensive methods (combining several *Hp* detection techniques), and one study used urease breath test (UBT). The mean age of all the subjects ranged from 13.00 to 50.2 years, and 51% of the participants were women. However, some information about age or sex was unclear. In this meta-analysis, 13 studies involving 1403 IBS patients and 11,770 non-IBS participants were included.

### 3.3. The Association between HPI and IBS

**Figure 3:**
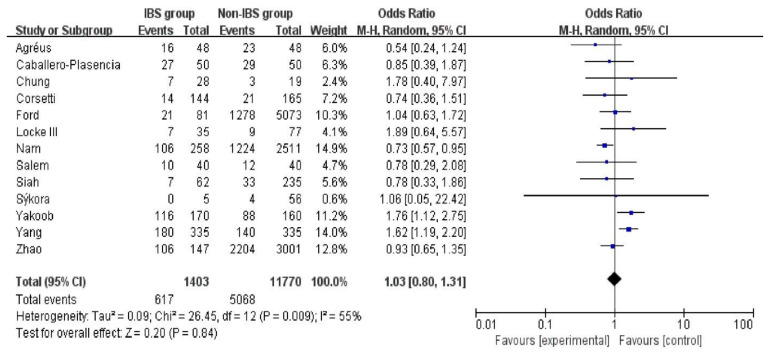
Forest plot of the overall association between HPI and IBS. M-H, Mantel–Haenszel; HPI, *Helicobacter pylori* infection; IBS, irritable bowel syndrome [14,16,27,28,29,30,31,32,33,34,35,36,37].

The association between HPI and IBS is depicted in a forest plot in Figure 3. The overall OR of the association between HPI and IBS was 1.03 (95% CI [0.80,1.31]; *p* = 0.84), which was nonsignificant. In addition, the heterogeneity (*p* = 0.009; I^2^ = 55%) was noteworthy. The RE model was applied in the calculation according to the value of heterogeneity.

### 3.4. Subgroup Analysis of the Association between HPI and IBS

The RE model was applied in all subgroup analyses. The results of all subgroup analyses are presented in Table 2. The detailed forest plots of all subgroup analyses are provided in Supplementary Material S4.

The OR of the correlation between HPI and IBS was 1.14 (95% CI [0.78,1.66]; *p* = 0.50; heterogeneity: *p* = 0.0007; I^2^ = 77%), 0.84 (95% CI [0.60,1.17]; *p* = 0.30; heterogeneity: *p* = 0.75; I^2^ = 0%), and 1.18 (95% CI [0.49,2.80]; *p* = 0.71; heterogeneity: *p* = 0.23; I^2^ = 29%) in Asia, Europe, and the other regions, respectively.

In the community-based population, nonsignificant increased incidence of HPI was observed in the IBS group compared with the non-IBS group (OR: 0.93; 95% CI [0.72,1.21]; *p* = 0.60; heterogeneity: *p* = 0.46; I^2^ = 0%). Same as the former, nonsignificant increased incidence of HPI was noted in IBS patients compared with non-IBS participants (OR: 1.09; 95% CI [0.74,1.58]; *p* = 0.67; heterogeneity: *p* = 0.002; I^2^ = 68%) in the hospital-based population.

According to the results of the Rome criteria group (OR: 1.04; 95% CI [0.78,1.39]; *p* = 0.80; heterogeneity: *p* = 0.006; I^2^ = 61%) and Manning criteria group (OR: 1.16; 95% CI [0.73,1.82]; *p* = 0.53; heterogeneity: *p* = 0.33; I^2^ = 0%), the incidence of HPI was no different from that in the IBS patients than that of the non-IBS participants. Moreover, the results of the other criteria group (1 included study; OR: 0.54; 95% CI [0.24,1.24]; *p* = 0.15) were the same.

In the immunological method group (OR: 0.88; 95% CI [0.67,1.16]; *p* = 0.37; heterogeneity: *p* = 0.63; I^2^ = 0%), RUT group (OR: 0.83; 95% CI [0.46,1.51]; *p* = 0.54; heterogeneity: *p* = 0.25; I^2^ = 23%), histological method group (OR: 1.19; 95% CI [0.51,2.78]; *p* = 0.68; heterogeneity: *p* = 0.04; I^2^ = 75%), and UBT group (one study included; OR: 1.04; 95% CI [0.63,1.72]; *p* = 0.88), the HPI was noted to be not correlated with IBS. Nevertheless, a positive correlation was observed in the comprehensive method group (OR: 1.61; 95% CI [1.19,2.18]; *p* = 0.002 < 0.05; heterogeneity: *p* = 0.79; I^2^ = 0%).

In addition, the OR for the correlation between HPI and IBS was 1.13 (95% CI [0.80,1.60]; *p* = 0.5; heterogeneity: *p* = 0.08; I^2^ = 45%) and 0.85 (95% CI [0.70,1.04]; *p* = 0.12; heterogeneity: *p* = 0.37; I^2^ = 6%) in the case–control study group and the cross-sectional study group, respectively.

According to the results mentioned above, only the factor of study design was identified as a source of heterogeneity (all I^2^ values in that factor were less than the pooled I^2^ of 55%). In addition, the factor of the *Hp* detection method impacted the correlation between HPI and IBS (The P-value of the comprehensive method group is 0.002 < 0.05).

### 3.5. Association between HPI and IBS after Studies with Defined Confounding Factors Were Excluded

**Figure 4:**
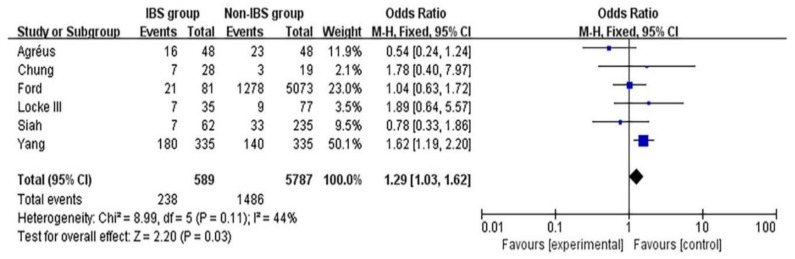
Forest plot of the adjusted association between HPI and IBS after the studies with defined confounding factors were excluded. M-H, Mantel–Haenszel; HPI, *Helicobacter pylori* infection; IBS, irritable bowel syndrome [16,27,28,32,33,35].

The adjusted OR was calculated after excluding studies with defined confounding factors (Figure 4). The FE model was adopted for calculation. We found that HPI was significantly associated with IBS (adjusted OR: 1.29; 95% CI [1.03,1.62]; *p* = 0.03 < 0.05; heterogeneity: *p* = 0.11; I^2^ = 44%).

### 3.6. Association between HPI and IBS of Different Subtypes

**Figure 5:**
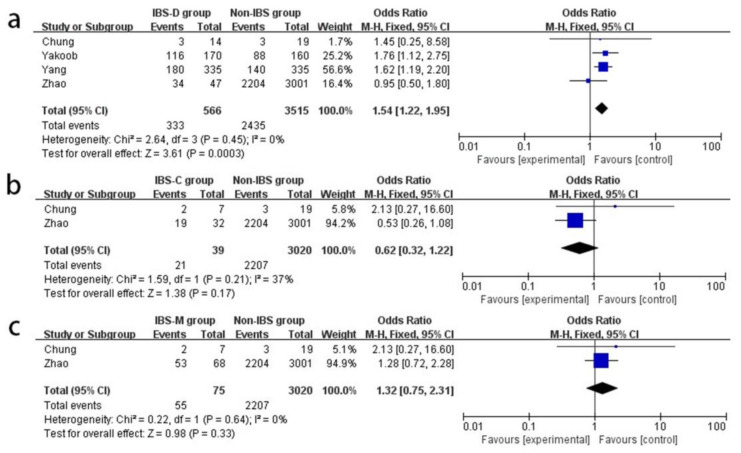
Forest plots of the associations between HPI and different subtypes of IBS. Forest plot of the association between HPI and IBS-D (**a**). Forest plot of the association between HPI and IBS-C (**b**). Forest plot of the association between HPI and IBS-M (**c**). M-H, Mantel–Haenszel; HPI, *Helicobacter pylori* infection; IBS-D, irritable bowel syndrome of diarrhea type; IBS-C, irritable bowel syndrome of constipation type; IBS-M, irritable bowel syndrome of mixed type [14,16,28,34].

As the heterogeneity of the results satisfied the criteria when applying the FE model, the FE model was adopted for calculation. The association between HPI and IBS-D is shown in a forest plot in Figure 5a. We found a positive association between HPI and IBS-D (OR: 1.54; 95% CI [1.22,1.95]; *p* = 0.0003 < 0.05; heterogeneity: *p* = 0.45; I^2^ = 0%). However, as shown in Figure 5b,c separately, HPI is not associated with IBS-C (OR: 0.62; 95% CI [0.32,1.22]; *p* = 0.17; heterogeneity: *p* = 0.21; I^2^ = 37%) or IBS-M (OR: 1.32; 95% CI [0.75,2.31]; *p* = 0.33; heterogeneity: *p* = 0.64; I^2^ = 0%).

### 3.7. Association between Cag A-Positive HPI and IBS

**Figure 6:**
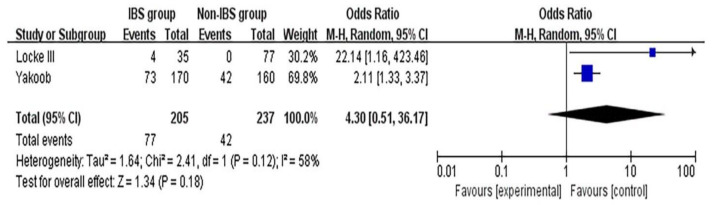
Forest plot of the association between Cag A-positive HPI and IBS. M-H, Mantel–Haenszel; HPI, *Helicobacter pylori* infection; IBS, irritable bowel syndrome; Cag A, cytotoxin-associated gene A [14,32].

The forest plot of this association is presented in Figure 6. The RE model was adopted for the calculation. Although a nonsignificant association between Cag A-positive HPI and IBS was found, the likelihood of Cag A-positive HPI in IBS patients is much higher than that of the non-IBS participants (OR: 4.3; 95% CI [0.51,36.17]; *p* = 0.18; heterogeneity: *p* = 0.12; I^2^ = 58%).

### 3.8. Publication Bias Assessment

**Figure 7:**
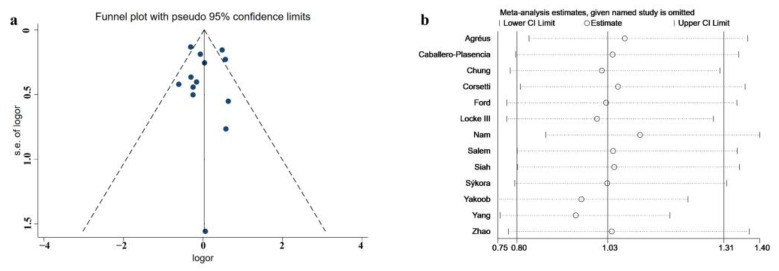
Funnel plot of publication bias assessment (**a**) and figure of sensitivity analysis (**b**) regarding overall association between HPI and IBS [14,16,27,28,29,30,31,32,33,34,35,36,37].

The funnel plot of the association between HPI and IBS is presented in Figure 7a. Egger’s test and Begg’s test (Supplementary Material S5) were performed to assess the asymmetry of the funnel plot. No significant publication bias was observed (Egger’s test *p* = 0.979; Begg’s test *p* = 0.855).

We did not assess the publication bias of the other associations between HPI and IBS because the numbers of included studies were less than ten.

### 3.9. Sensitivity Analysis

The results of the sensitivity analysis of the association between HPI and IBS are presented in Figure 7b. The results support a stable association between HPI and IBS.

The robustness of the association between Cag A-positive HPI and IBS was not assessed because of the limited number of studies included. The robustness of other associations between HPI and IBS was not assessed because the heterogeneity was acceptable.

## 4. Discussion

A large-scale meta-analysis was conducted in the present investigation, which included 13,173 participants (1403 IBS patients and 11,770 non-IBS participants) from 13 studies to examine the link between HPI and IBS. Some previous meta-analyses on this topic included 1862 to 7143 participants from eight to ten studies [18,19,20]. The overall OR of this meta-analysis was 1.03 (95% CI [0.80,1.31]; *p* = 0.84), suggesting that the likelihood of HPI in IBS patients is relatively higher than that of the non-IBS participants but not statistically significant, implying that HPI may not associate with IBS.

*Hp* detection method might impact the correlation between HPI and IBS according to the results of subgroup analyses. The sensitivity and specificity of each *Hp* detection method were quite different, which may reduce the accuracy of the results. For instance, serological tests for *Hp* may include the patients who have recovered from HPI, which may lead to an overestimate of HPI cases [38]. Additionally, the salivary assay of *Hp* may underreport the HPI cases because its sensitivity and specificity were no more than 81% [38,39]. Thus, such differences in different *Hp* detection methods may lead to an unpredictable impact on associations between HPI and IBS. Nested PCR may have been the best detection method because the sensitivity and specificity were both 100% when targeting the highly conserved gene of *Hp* [40,41]. However, its wide application is limited by high service costs and complex operations. It is reported that a more feasible solution is to adopt comprehensive methods that combine the results of more than two *Hp* detection methods and form a conclusion after comparing the results of each method to acquire high sensitivity and specificity in *Hp* detection [40]. Actually, in daily clinical practice, UBT is a practical and feasible solution with high accuracy, wide application, and relatively low cost [42,43]. RUT is also widely used as a screening test in clinical practice, which is economical, fast, and simple [44]. Therefore, the significant positive correlation between HPI and IBS in the comprehensive method group may be accurate. The factor of study design was identified as a major heterogeneity source according to the results of subgroup analyses. Cross-sectional and case-control studies are easily impacted by information bias, such as recall bias, reporting bias, and exposure suspicion bias. Thus, the inherent information bias in the study designs may cause heterogeneity. Moreover, the case-control study design is a more suitable study design for identifying disease risk factors than the cross-sectional study design, and evidence from case-control studies has a higher level.

Actually, the overall OR of this meta-analysis was not significantly different from those of published meta-analyses [18,19,20]. However, in some included studies, we found that participants exhibited other gastrointestinal symptoms [29] or suffered from other gastrointestinal disorders, such as dyspepsia [12,30,34,37], non-erosive reflux disease [36], and chronic diarrhea [14]. *Hp* might be a risk factor for these gastrointestinal disorders, according to some studies [45,46,47]. Moreover, in one included study, all of the participants were children [31]. These studies were included in the published meta-analyses, which may have impacted their results and conclusions. To ascertain the link between HPI and IBS, the adjusted OR was calculated after excluding these studies. The adjusted results indicated that HPI was significantly related to IBS (pooled OR: 1.29; 95% CI [1.03,1.62]; *p* = 0.03 < 0.05; heterogeneity: *p* = 0.11; I^2^ = 44%). In addition, Liang and his colleagues reported that the IBS prevalence rate in the *Hp*-positive cohort was significantly higher than that in the *Hp*-negative cohort (log-rank test, *p* < 0.001), which may support this finding [15]. The finding demonstrated that the confounding factors might make us underestimate the association between HPI and IBS, encouraging researchers to design high-quality prospective studies to investigate it further.

Gut infection is one of the most crucial mechanisms in the pathogenesis of IBS, causing alterations in gut permeability and inflammation, leading to post-inflammatory neuroplastic changes and visceral hyperalgesia [6]. *Hp* has been acknowledged to be an important pathogen in gastrointestinal inflammation, which may cause edema (alteration of mucosa permeability) and inflammation of the gastrointestinal mucosa [9,46]. Cag A is a major virulence factor of *Hp* that can aggravate the dysfunction of the intestinal epithelium barrier, implying the potential impact of Cag A on the lower gastrointestinal tract [48]. Yakoob et al. reported that Cag A-positive HPI promotes the development of IBS-D [14]. Interestingly, we found that the likelihood of Cag A-positive HPI in IBS patients is much higher than that of the non-IBS participants, although the association is nonsignificant. Moreover, we found a positive association between HPI and IBS-D. Based on these evidence, we speculated that Cag A-positive HPI might be a potential risk factor for IBS-D, while whether Cag A-positive HPI can promote the overall incidence of HPI remains unclear. However, Cag A usually affects the upper gastrointestinal tract, such as the gastric and duodenum, by inflammation instead of the lower gastrointestinal tract [49,50,51]. Although there are many studies showing that Cag A is associated with extra-gastroduodenal diseases such as aortic endothelial inflammation and atherosclerosis, cardiac syndrome x, and Behçet’s disease [52,53,54], it is still doubtful whether Cag A can affect the lower gastrointestinal tract. Moreover, studies have reported that pathogen infection may cause an inflammatory cascade in IBS. The primary pathogenic mechanisms of gut microbiota include increased adhesion of bacteria to the gut mucosa, immune system evasion, and inhibition of the host immune response [55]. Pathogens can activate signaling pathways to recruit eosinophils and mast cells, thus resulting in the high expression of cytokine proteins, histamines, and serotonin, which stimulate nerves, causing pain and muscle spasms [6,56,57]. On the one hand, some studies have reported that *Hp* might promote the inflammatory response in the lower gastrointestinal tract [58,59]. On the other hand, some researchers found that HPI was a protective factor for inflammatory bowel disease [60,61]. Thus, it remains doubtful whether *Hp* can affect the lower gastrointestinal tract by a postinfectious inflammatory response like other intestinal bacteria. More experimental studies are suggested to address this issue. It is reported that *Hp*-infected IBS patients were more likely to exhibit abdominal symptoms after stimulation with a rectal stimulator, unlike IBS patients who did not seem to be infected by *Hp*, implying that *Hp* causes neuroplastic changes and visceral hyperalgesia [19,62].

It is still unknown whether the eradication of *Hp* can cure IBS or relieve IBS symptoms. Some studies have shown that IBS patients did not benefit from *Hp* eradication therapy [16,17]. However, a meta-analysis reported that anti-*Hp* treatment effectively relieved symptoms of IBS [63]. According to our findings, the associations between HPI and different subtypes of IBS may be different. Moreover, the associations between HPI of different virulence factors and IBS may be different. Therefore, not all patients with HPI require *Hp* eradication therapy and *Hp* eradication therapy should be targeted at specific IBS subtypes or *Hp* subtypes. Well-designed randomized controlled trials are required to investigate the therapeutic effect of *Hp* eradication therapy in IBS patients based on the current evidence.

The strengths of this study are as follows. First, this meta-analysis is the most extensive study investigating the relationship between HPI and IBS. Second, compared with the published meta-analysis, our study involved more baseline information and more comprehensive factors to explore the sources of heterogeneity. Third, we calculated the adjusted OR after excluding the studies with confounding factors defined by us to obtain a more accurate association between HPI and IBS. Fourth, we first studied the association between HPI and different subtypes of IBS. Fifth, we first examined the relationship between Cag A-positive HPI and IBS. Therefore, the results of this study are more comprehensive, more reliable, more accurate, and more robust than those of previously published meta-analyses.

There are several limitations in this meta-analysis that should be noted. First, potential confounding bias still exists. We did not consider the impacts of age, sex, or medical history. Second, various kinds of IBS diagnostic criteria and *Hp* detection methods were applied in the included studies. Due to the various sensitivities and specificities of IBS diagnostic criteria and *Hp* detection methods, potential observation bias may be present. For instance, *Hp* serological test may cause a pseudo-positive result because of the past-infection patients. Third, there is markable heterogeneity that should be treated with caution. The heterogeneity of the overall association between HPI and IBS is noteworthy, although we explored its sources considering five factors via subgroup analysis and identified the primary source of the heterogeneity as study design; the high heterogeneity existed in the relationship between Cag A-positive HPI and IBS. Fourth, the relationship between HPI and different subtypes of IBS is different, which means the overall relationship between HPI and IBS may be impacted by this difference. It is suggested to investigate the relationship between HPI and different subtypes of IBS separately instead of simply studying the overall relationship between HPI and IBS.

## 5. Conclusions

The overall likelihood of HPI in IBS patients is relatively higher than that of non-IBS participants, but the association is not statistically significant. After the studies with defined confounding factors were excluded, we found that HPI was significantly associated with IBS, suggesting that we may underestimate the association between HPI and IBS. In conclusion, based on the current evidence, HPI is not significantly associated with IBS, albeit we may underestimate it. Moreover, the association between HPI and different subtypes of IBS is different. We observed a positive association between HPI and IBS-D. Furthermore, the likelihood of Cag-A positive HPI in IBS patients was much higher compared with non-IBS participants, and the current experimental evidence has also supported that Cag A can promote the development of IBS-D. Additional high-quality prospective studies are still required to investigate the relationship between HPI and IBS. Moreover, the specific role of Cag A in the pathogenic mechanism of IBS remains unclear, and relevant experimental studies are suggested to study this issue.

## Figures and Tables

**Table 1:** The characteristics of the studies included in meta-analysis.

Author	Year	Region	Design of Study	Study Population	IBS Diagnostic Criteria	*Hp* Detection Method	Mean Age ± SD, Median Age or Age Range (Year)	Sex (Female/Male)	OR	IBS Patients’ Status	Non-IBS Participants’ Status	NOS/AHRQ
Agréus et al. [33]	1995	Sweden	Case-control	Community-based	Diagnostic criteria designed by authors	Immunological method (serum sample + HM-CAP immunoassay)	Mean age: 48 Age range: 22–80 (all participants of the study)	96/54 (all participants of the study)	0.54	IBS alone	Participants without abdominal or bowel symptoms	6
Caballero-Plasencia et al. [37]	1999	Spain	Case-control	Hospital-based	Rome I	Immunological method (antibody-IgG for *Hp*)	IBS: 34.1 ± 7.4 Non-IBS: 35.6 ± 5.6	IBS: 25/25 Non-IBS: 25/25	0.85	22 IBS alone, 28 IBS + FD	Free of digestive symptoms or systemic disease	7
Chung et al. [28]	2016	Taiwan	Case-control	Hospital-based	Rome III	RUT (gastric biopsy specimens + RUT)	IBS: 44.84 ± 14.66 Non-IBS: 42.66 ± 11.16	IBS: 16/12 Non-IBS: 12/7	1.78	IBS alone	Healthy people	6
Corsetti et al. [30]	2004	Belgium	Case-control	Hospital-based	Rome II	Histology method (gastric biopsy specimens + cresyl violet staining)	IBS: 42 ± 1.2 Non-IBS: 42 ± 1.1	IBS: 108/36 Non-IBS: 99/66	0.74	IBS + FD	FD patients	6
Ford et al. [27]	2012	UK	Cross-sectional *	Community-based	Manning	UBT	IBS: 45.2 ± 2.9 Non-IBS: 45.3 ± 2.9	IBS: 69/12 Non-IBS:2707/2366	1.04	IBS alone	General population without meeting criteria for dyspepsia or IBS	7
Locke III et al. [32]	2000	USA	Cross-sectional	Community-based	Manning	Immunological method (serum sample + ELISA)	Median age IBS: 31 Non-IBS: 39	IBS: 25/10 Non-IBS: 38/39	1.89	IBS alone	Healthy people	7
Nam et al. [36]	2013	Korea	Cross-sectional	Hospital-based	Rome III	RUT (gastric biopsy specimens + RUT)	IBS: 45.3 ± 8.6 Non-IBS: 50.2 ± 9.9	IBS: 89/169 Non-IBS: 998/1513	0.73	IBS alone, IBS + NERD, IBS + erosive esophagitis	Some participants were NERD patients	6
Salem et al. [29]	2019	Egypt	Case-control	Hospital-based	Rome III	Immunological method (fresh stool specimens + ELISA)	IBS: 28 ± 9.5 Non-IBS: 33.5 ± 13.9	NR	0.78	IBS alone	Patients with gastrointestinal symptoms but do not fulfill the IBS criteria	6
Siah et al. [35]	2016	Singapore	Cross-sectional	Community-based	Rome III	Immunological method (saliva samples + ELISA)	Over 21 years old	IBS: 40/22 Non-IBS:139/96	0.78	IBS alone	General population without reporting clear health status	6
Sýkora et al. [31]	2016	Czech Republic	Case-control	Hospital-based	Rome III	Comprehensive method (stool antigen test, *Hp* culture or biopsy-based diagnostic tests)	Median age IBS: NR Non-IBS: 13	IBS: NR Non-IBS: 32/25	1.06	Children patients	Children without any signs of gastrointestinal conditions	6
Yakoob et al. [14]	2012	Pakistan	Case-control	Hospital-based	Rome III	Histology method (gastric biopsy specimens + histopathology test)	IBS: 40 ± 15 Non-IBS: 42 ± 14	IBS: 54/116 Non-IBS: 54/106	1.76	IBS alone	Chronic diarrhea	6
Yang et al. [16]	2017	China	Case-control	Hospital-based	Rome III	Comprehensive method (gastric biopsy specimens + RUT and ^14^C-UBT)	IBS: 40.53 ± 13.27 Non-IBS: 41.96 ± 12.89	IBS: 158/177 Non-IBS: 183/152	1.62	IBS alone	Healthy people	6
Zhao et al. [34]	2010	China	Cross-sectional	Community-based	Rome II	Immunological method (blood sample + ELISA)	All participants: 42.5 ± 15.2	All participants: 8393/7685	0.93	IBS alone, IBS + other digestive disease	Participants with poor health or other digestive diseases	7

RUT, rapid urease test; UBT, urease breath test; ELISA, enzyme-linked immunosorbent assay; HM-CAP immunoassay, high molecular weight cell-associated protein immunoassay; NOS, Newcastle-Ottawa Scale; AHRQ, Agency for Healthcare Research and Quality; BDQ, bowel disease questionnaire; NHIRD, National Health Insurance Research Database; IBS, irritable bowel syndrome; FD, functional dyspepsia; *Hp*, *Helicobacter pylori*; NERD, non-erosive reflux disease; FGDs, functional gastroduodenal disorders; NR, not reported; * an cross-sectional investigation of a cohort study.

**Table 2:** Subgroup analysis of the association between HPI and IBS.

	No. of Studies	No. of Participants	The Association between HPI and IBS			Heterogeneity		Subgroup Differences
			Pooled OR	Confidence Interval	*p* Value	I ²	*p* Value	*p* Value
**Region**								0.45
Asia	6	7261	1.14	[0.78, 1.66]	0.5	77%	0.0007	
Europe	5	5720	0.84	[0.60, 1.17]	0.3	0%	0.75	
Other regions	2	192	1.18	[0.49, 2.80]	0.71	29%	0.23	
**Study population**								0.52
Community-based	5	8807	0.93	[0.72, 1.21]	0.6	0%	0.46	
Hospital-based	8	4366	1.09	[0.74, 1.58]	0.67	68%	0.002	
**IBS diagnostic criteria**								0.28
Rome	10	7811	1.04	[0.78, 1.39]	0.8	61%	0.006	
Manning	2	5266	1.16	[0.73, 1.82]	0.53	0%	0.33	
Other criteria	1	96	0.54	[0.24, 1.24]	0.15	-	-	
***Hp*** **detection method**								0.05
Immunological method	6	3833	0.88	[0.67, 1.16]	0.37	0%	0.63	
Histological method	2	639	1.19	[0.51, 2.78]	0.68	75%	0.04	
RUT	2	2816	0.83	[0.46, 1.51]	0.54	23%	0.25	
Comprehensive method	2	731	1.61	[1.19, 2.18]	0.002	0%	0.79	
UBT	1	5154	1.04	[0.63, 1.72]	0.88	-	-	
**Study design**								0.18
Cross-sectional study	5	11,480	0.85	[0.70, 1.04]	0.12	6%	0.37	
Case-control study	8	1693	1.13	[0.80, 1.60]	0.5	45%	0.08	

*Hp, Helicobacter pylori*; IBS, irritable bowel syndrome; RUT, rapid urease test; UBT, urease breath test.

## Data Availability

The detailed data of this study are available from the corresponding author.

## Supplementary Materials

The following supporting information can be downloaded at: https://www.mdpi.com/article/10.3390/medicina58081035/s1, Supplementary Material S1: The detailed search strategies used in EMBASE and the Cochrane Library. Supplementary Material S2: The results of quality assessment for each study included in meta-analysis. Supplementary Material S3: The detailed numbers of all patients and participants and the funding information of all included studies. Supplementary Material S4: The detailed forest plots of all subgroup analyses. Supplementary Material S5: The plots of Egger’s test and Begg’s test.
