# Supplementary material for: Helicobacterpylori Infection—A Risk Factor for Irritable Bowel Syndrome? An Updated Systematic Review and Meta-Analysis
Source: Medicina (Kaunas). 2022 Aug 2;58(8):1035. doi: 10.3390/medicina58081035 (PMC9413972; doi:10.3390/medicina58081035)
Supplement: Supplementary file 1 [file medicina-58-01035-s001.zip › Supplementary Material S1.pdf]

Supplementary Material S1. Search strategies for EMBASE and the Cochrane Library.

EMBASE Search Strategy:

#1 ‘Helicobacter pylori’

#2 ‘Helicobacter nemestrinae’

#3 ‘Campylobacter pylori’

#4 ‘Campylobacter pylori subsp. Pylori’

#5 ‘Campylobacter pyloridis’

#6 ‘H. pylori’

#7 ‘pylori’

#8 ‘Helicobacter’

#9 ‘Irritable Colon’

#10 ‘Mucous Colitis’

#11 ‘Mucous Colitides’

#12 ‘Irritable Bowel Syndrome’

#13 ‘Irritable Bowel Syndromes’

#14 ‘IBS’

#15 #1 OR #2 OR #3 OR #4 OR #5 OR #6 OR #7 OR #8

#16 #9 OR #10 OR #11 OR #12 OR #13 OR #14

#17 #15 AND #16

Cochrane Library Search Strategy:

#1 MeSH descriptor： [Helicobacter pylori] explode all trees

#2 (Helicobacter nemestrinae):ti,ab,kw

#3 (Campylobacter pylori):ti,ab,kw

#4 (Campylobacter pylori subsp. Pylori):ti,ab,kw

#5 (Campylobacter pyloridis):ti,ab,kw

#6 (H. pylori):ti,ab,kw

#7 (pylori):ti,ab,kw

#8 (Helicobacter):ti,ab,kw

#9 MeSH descriptor： [Irritable Bowel Syndrome] explode all trees

#10 (Irritable Bowel Syndromes):ti,ab,kw

#11 (Irritable Colon):ti,ab,kw

#12 (Irritable Colons):ti,ab,kw

#13 (Mucous Colitis):ti,ab,kw

#14 (IBS):ti,ab,kw

#15 #1 OR #2 OR #3 OR #4 OR #5 OR #6 OR #7 OR #8

#16 #9 OR #10 OR#11 OR #12 OR #13 OR #14

#17 #15 AND #16
