# Supplementary material for: Helicobacterpylori Infection—A Risk Factor for Irritable Bowel Syndrome? An Updated Systematic Review and Meta-Analysis
Source: Medicina (Kaunas). 2022 Aug 2;58(8):1035. doi: 10.3390/medicina58081035 (PMC9413972; doi:10.3390/medicina58081035)
Supplement: Supplementary file 1 [file medicina-58-01035-s001.zip › Supplementary Material S2.pdf]

Supplementary Materials S2. The results of quality evaluation of the included study.

1. The quality evaluation of case-control study (NOS scale).

Study	Selection (0~4)	Comparability (0~2)	Exposure (0~3)	Total number of stars
Agréus	3	1	2	6
Caballero-Plasencia	2	2	3	7
Chung	3	1	2	6
Corsetti	2	1	3	6
Salem	2	1	3	6
Sýkora	2	1	3	6
Yakoob	2	1	3	6
Yang	2	1	3	6

2. The quality evaluation of cross-sectional study (AHRQ scale)

	Ford	Locke III	Nam	Siah	Zhao
1) Define the source of information (survey, record review)	Yes	Yes	Yes	Yes	Yes
2) List inclusion and exclusion criteria for exposed and unexposed subjects (cases and controls) or refer to previous publications	Yes	Yes	Yes	Yes	Yes
3) Indicate time period used for identifying patients	Yes	Unlear	Yes	Unclear	Yes
4) Indicate whether or not subjects were consecutive if not population-based	Yes	Yes	Yes	Yes	Yes
5) Indicate if evaluators of subjective components of study were masked to other aspects of the status of the participants	Yes	Yes	Yes	Yes	Yes
6) Describe any assessments undertaken for quality assurance purposes (e.g., test/retest of primary outcome measurements)	Yes	Yes	Unclear	Yes	Yes
7) Explain any patient exclusions from analysis	No	Yes	Unclear	Unclear	Unclear
8) Describe how confounding was assessed and/or controlled.	Unclear	Unclear	Unclear	Unclear	Unclear
9) If applicable, explain how missing data were handled in the analysis	No	No	No	No	No
10) Summarize patient response rates and completeness of data collection	Yes	Yes	Yes	Yes	Yes
11) Clarify what follow-up, if any,	Unclear	Unclear	Unclear	Unclear	Unclear

was expected and the percentage of patients for which incomplete data or follow-up was obtained					
Total items were met (total number of stars)	7	7	6	6	7
