# Supplementary material for: Helicobacterpylori Infection—A Risk Factor for Irritable Bowel Syndrome? An Updated Systematic Review and Meta-Analysis
Source: Medicina (Kaunas). 2022 Aug 2;58(8):1035. doi: 10.3390/medicina58081035 (PMC9413972; doi:10.3390/medicina58081035)
Supplement: Supplementary file 1 [file medicina-58-01035-s001.zip › Supplementary Material S3.pdf]

Supplementary Material S3. The detailed number of the patients and participants involved in meta-analysis and the funding information of included studies.

	IBS group		Non-IBS group		Funding information
Author	No. of all patients	No. of HPI	No. of all participants	No. of HPI	Not reported
Agréus	48	16	48	23	Not reported
Caballero-Plasencia	50	27	50	29	Not reported
Chung	28 （14 IBS-D, 7 IBS-C and 7 IBS-M）	7 （3 HPI in IBS-D, 2 HPI in IBS-C and 2 HPI in IBS-M）	19	3	Not reported
Corsetti	144	14	165	21	Not reported
Ford	81	21	5073	1278	University partly funded by an unrestricted donation by Astra Zeneca, and has received consultant ' s and speaker ' s bureau fees
Locke III	35	7 (4 Cag A-positive participants)	77	9 (0 Cag A-positive participants）	Not reported
Nam	258	106	2511	1224	This study was supported by a grant (No. NCC 0810200-1) from the National Cancer Center, Korea.
Salem	40	10	40	12	Not reported
Siah	62	7	235	33	Not reported
Sýkora	5	0	56	4	Not reported
Yakoob	170 (170 IBS-D)	116 (73 Cag A-positive participants)	160	88 (42 Cag A-positive participants)	Not reported
Yang	335 (335 IBS-D)	180	335	140	Not reported
Zhao	147 （47 IBS-D, 32 IBS-C and 68 IBS-M）	106 （34 HPI in IBS-D, 19 HPI in IBS-C and 53 HPI in IBS-M）	3001	2204	This study was supported by AstraZen-eac R&D, Mo¨lndal, Sweden.
