# Supplementary material for: Helicobacterpylori Infection—A Risk Factor for Irritable Bowel Syndrome? An Updated Systematic Review and Meta-Analysis
Source: Medicina (Kaunas). 2022 Aug 2;58(8):1035. doi: 10.3390/medicina58081035 (PMC9413972; doi:10.3390/medicina58081035)

Supplementary Material S4. The forest plots of subgroup analyses of the associations between HPI and IBS

1.The forest plot of subgroup analysis stratified by region

Study or Subgroup	IBS group Events	IBS group Total	Non-IBS group Events	Non-IBS group Total	Weight	Odds Ratio M-H, Random, 95% CI
**1.2.1 Asia**						
Chung	7	28	3	19	2.4%	1.78 [0.40, 7.97]
Nam	106	258	1224	2511	14.9%	0.73 [0.57, 0.95]
Siah	7	62	33	235	5.6%	0.78 [0.33, 1.86]
Yakoob	116	170	88	160	11.2%	1.76 [1.12, 2.75]
Yang	180	335	140	335	14.0%	1.62 [1.19, 2.20]
Zhao	106	147	2204	3001	12.8%	0.93 [0.65, 1.35]
**Subtotal (95% CI)**		**1000**		**6261**	**60.8%**	**1.14 [0.78, 1.66]**
Total events	522		3692			
Heterogeneity: Tau² = 0.15; Chi² = 21.31, df = 5 (P = 0.0007); I² = 77%						
Test for overall effect: Z = 0.67 (P = 0.50)						
**1.2.2 Europe**						
Agréus	16	48	23	48	6.0%	0.54 [0.24, 1.24]
Caballero-Plasencia	27	50	29	50	6.3%	0.85 [0.39, 1.87]
Corsetti	14	144	21	165	7.2%	0.74 [0.36, 1.51]
Ford	21	81	1278	5073	10.3%	1.04 [0.63, 1.72]
Sýkora	0	5	4	56	0.6%	1.06 [0.05, 22.42]
**Subtotal (95% CI)**		**328**		**5392**	**30.4%**	**0.84 [0.60, 1.17]**
Total events	78		1355			
Heterogeneity: Tau² = 0.00; Chi² = 1.91, df = 4 (P = 0.75); I² = 0%						
Test for overall effect: Z = 1.03 (P = 0.30)						
**1.2.3 Other region**						
Locke III	7	35	9	77	4.1%	1.89 [0.64, 5.57]
Salem	10	40	12	40	4.7%	0.78 [0.29, 2.08]
**Subtotal (95% CI)**		**75**		**117**	**8.7%**	**1.18 [0.49, 2.80]**
Total events	17		21			
Heterogeneity: Tau² = 0.12; Chi² = 1.42, df = 1 (P = 0.23); I² = 29%						
Test for overall effect: Z = 0.37 (P = 0.71)						
**Total (95% CI)**		**1403**		**11770**	**100.0%**	**1.03 [0.80, 1.31]**
Total events	617		5068			
Heterogeneity: Tau² = 0.09; Chi² = 26.45, df = 12 (P = 0.009); I² = 55%						
Test for overall effect: Z = 0.20 (P = 0.84)						
Test for subgroup differences: Chi² = 1.59, df = 2 (P = 0.45), I² = 0%						

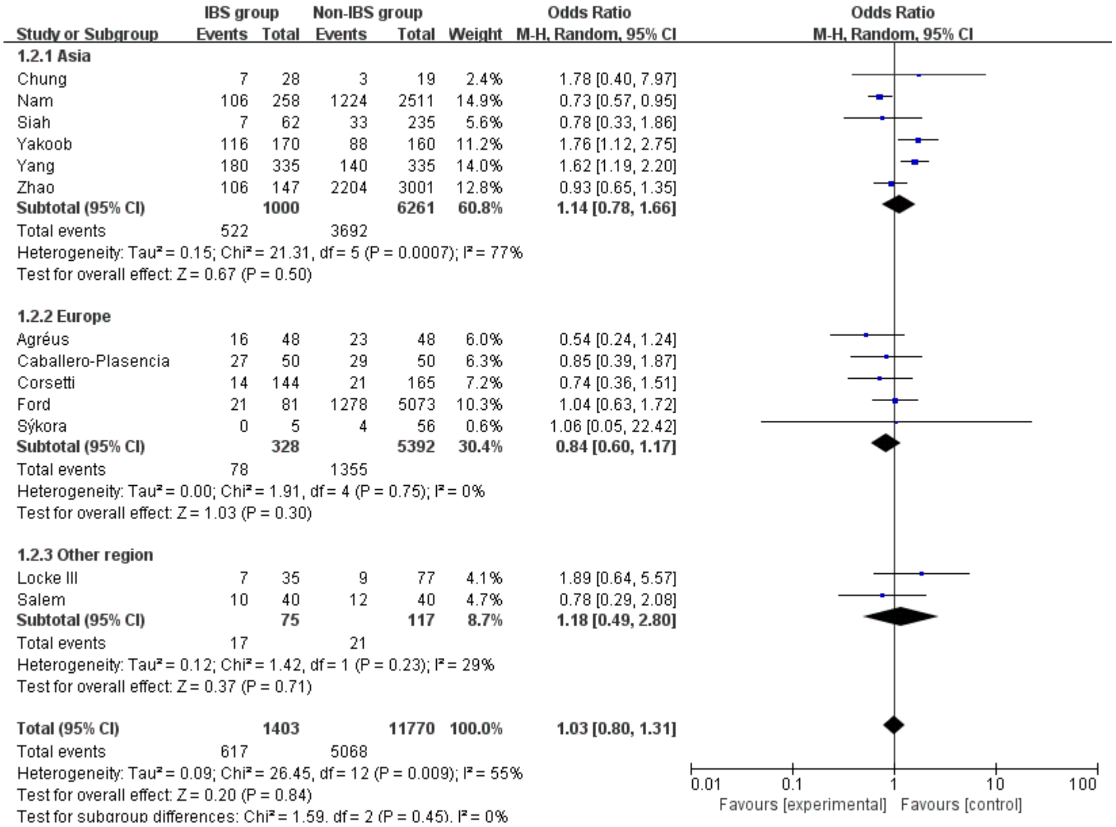

2.The forest plot of subgroup analysis stratified by IBS criteria

Study or Subgroup	IBS group Events	IBS group Total	Non-IBS group Events	Non-IBS group Total	Weight	Odds Ratio M-H, Random, 95% CI
**1.4.1 Rome**						
Caballero-Plasencia	27	50	29	50	6.3%	0.85 [0.39, 1.87]
Chung	7	28	3	19	2.4%	1.78 [0.40, 7.97]
Corsetti	14	144	21	165	7.2%	0.74 [0.36, 1.51]
Nam	106	258	1224	2511	14.9%	0.73 [0.57, 0.95]
Salem	10	40	12	40	4.7%	0.78 [0.29, 2.08]
Siah	7	62	33	235	5.6%	0.78 [0.33, 1.86]
Sýkora	0	5	4	56	0.6%	1.06 [0.05, 22.42]
Yakoob	116	170	88	160	11.2%	1.76 [1.12, 2.75]
Yang	180	335	140	335	14.0%	1.62 [1.19, 2.20]
Zhao	106	147	2204	3001	12.8%	0.93 [0.65, 1.35]
**Subtotal (95% CI)**		**1239**		**6572**	**79.6%**	**1.04 [0.78, 1.39]**
Total events	573		3758			
Heterogeneity: Tau² = 0.10; Chi² = 22.92, df = 9 (P = 0.006); I² = 61%						
Test for overall effect: Z = 0.26 (P = 0.80)						
**1.4.4 Manning**						
Ford	21	81	1278	5073	10.3%	1.04 [0.63, 1.72]
Locke III	7	35	9	77	4.1%	1.89 [0.64, 5.57]
**Subtotal (95% CI)**		**116**		**5150**	**14.4%**	**1.16 [0.73, 1.82]**
Total events	28		1287			
Heterogeneity: Tau² = 0.00; Chi² = 0.97, df = 1 (P = 0.33); I² = 0%						
Test for overall effect: Z = 0.62 (P = 0.53)						
**1.4.5 Other criteria**						
Agréus	16	48	23	48	6.0%	0.54 [0.24, 1.24]
**Subtotal (95% CI)**		**48**		**48**	**6.0%**	**0.54 [0.24, 1.24]**
Total events	16		23			
Heterogeneity: Not applicable						
Test for overall effect: Z = 1.45 (P = 0.15)						
**Total (95% CI)**		**1403**		**11770**	**100.0%**	**1.03 [0.80, 1.31]**
Total events	617		5068			
Heterogeneity: Tau² = 0.09; Chi² = 26.45, df = 12 (P = 0.009); I² = 55%						
Test for overall effect: Z = 0.20 (P = 0.84)						
Test for subgroup differences: Chi² = 2.54, df = 2 (P = 0.28), I² = 21.2%						

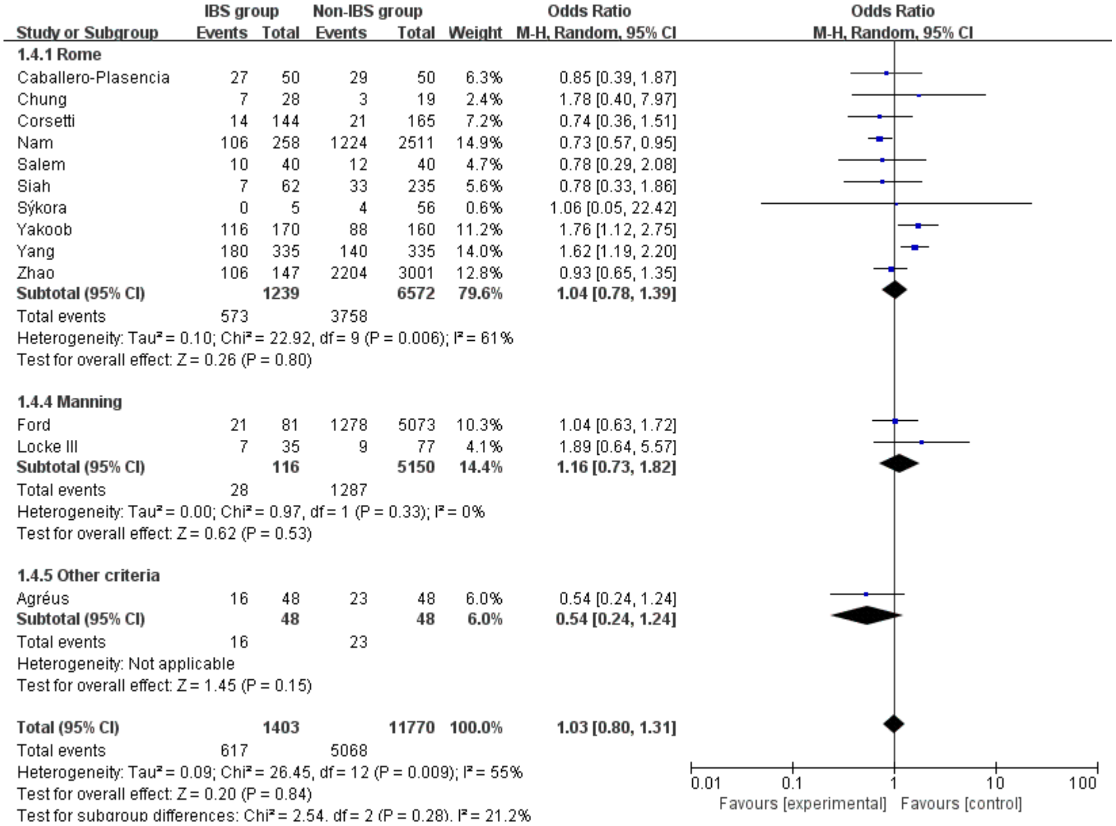

3.The forest plot of subgroup analysis stratified by *Hp* detection method

Study or Subgroup	IBS group Events	IBS group Total	Non-IBS group Events	Non-IBS group Total	Weight	Odds Ratio M-H, Random, 95% CI
**1.5.1 Immunological method**						
Agréus	16	48	23	48	6.0%	0.54 [0.24, 1.24]
Caballero-Plasencia	27	50	29	50	6.3%	0.85 [0.39, 1.87]
Locke III	7	35	9	77	4.1%	1.89 [0.64, 5.57]
Salem	10	40	12	40	4.7%	0.78 [0.29, 2.08]
Siah	7	62	33	235	5.6%	0.78 [0.33, 1.86]
Zhao	106	147	2204	3001	12.8%	0.93 [0.65, 1.35]
**Subtotal (95% CI)**		**382**		**3451**	**39.4%**	**0.88 [0.67, 1.16]**
Total events	173		2310			
Heterogeneity: Tau² = 0.00; Chi² = 3.47, df = 5 (P = 0.63); I² = 0%						
Test for overall effect: Z = 0.90 (P = 0.37)						
**1.5.2 Histological method**						
Corsetti	14	144	21	165	7.2%	0.74 [0.36, 1.51]
Yakoob	116	170	88	160	11.2%	1.76 [1.12, 2.75]
**Subtotal (95% CI)**		**314**		**325**	**18.4%**	**1.19 [0.51, 2.78]**
Total events	130		109			
Heterogeneity: Tau² = 0.28; Chi² = 4.04, df = 1 (P = 0.04); I² = 75%						
Test for overall effect: Z = 0.41 (P = 0.68)						
**1.5.3 RUT**						
Chung	7	28	3	19	2.4%	1.78 [0.40, 7.97]
Nam	106	258	1224	2511	14.9%	0.73 [0.57, 0.95]
**Subtotal (95% CI)**		**286**		**2530**	**17.2%**	**0.83 [0.46, 1.51]**
Total events	113		1227			
Heterogeneity: Tau² = 0.09; Chi² = 1.30, df = 1 (P = 0.25); I² = 23%						
Test for overall effect: Z = 0.62 (P = 0.54)						
**1.5.4 Comprehensive method**						
Sýkora	0	5	4	56	0.6%	1.06 [0.05, 22.42]
Yang	180	335	140	335	14.0%	1.62 [1.19, 2.20]
**Subtotal (95% CI)**		**340**		**391**	**14.6%**	**1.61 [1.19, 2.18]**
Total events	180		144			
Heterogeneity: Tau² = 0.00; Chi² = 0.07, df = 1 (P = 0.79); I² = 0%						
Test for overall effect: Z = 3.07 (P = 0.002)						
**1.5.5 UBT**						
Ford	21	81	1278	5073	10.3%	1.04 [0.63, 1.72]
**Subtotal (95% CI)**		**81**		**5073**	**10.3%**	**1.04 [0.63, 1.72]**
Total events	21		1278			
Heterogeneity: Not applicable						
Test for overall effect: Z = 0.15 (P = 0.88)						
**Total (95% CI)**		**1403**		**11770**	**100.0%**	**1.03 [0.80, 1.31]**
Total events	617		5068			
Heterogeneity: Tau² = 0.09; Chi² = 26.45, df = 12 (P = 0.009); I² = 55%						
Test for overall effect: Z = 0.20 (P = 0.84)						
Test for subgroup differences: Chi² = 9.52, df = 4 (P = 0.05), I² = 58.0%						

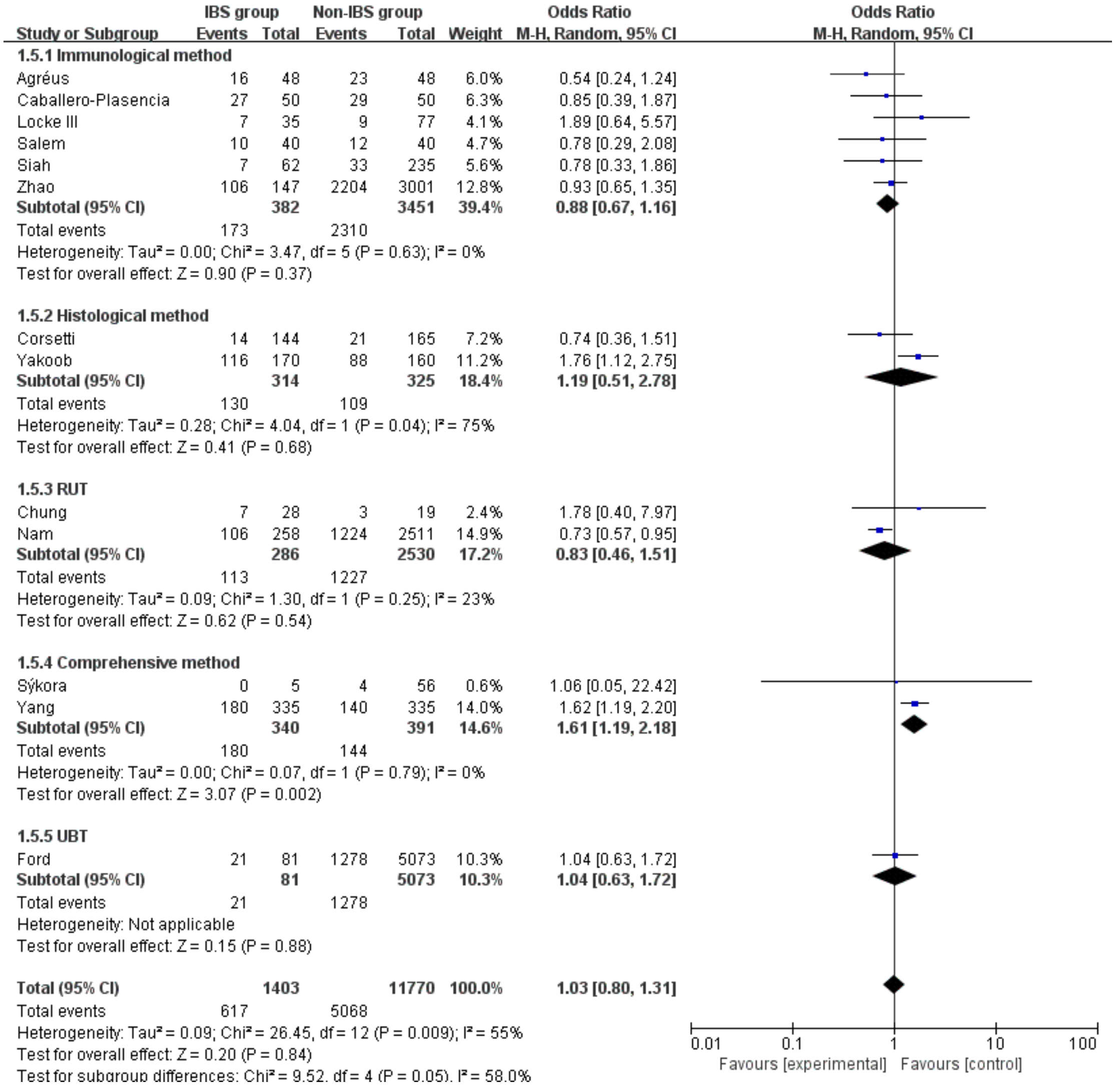

4.The forest plot of subgroup analysis stratified by study population

Study or Subgroup	IBS group Events	IBS group Total	Non-IBS group Events	Non-IBS group Total	Weight	Odds Ratio M-H, Random, 95% CI
**1.3.1 Community-based**						
Agréus	16	48	23	48	6.0%	0.54 [0.24, 1.24]
Ford	21	81	1278	5073	10.3%	1.04 [0.63, 1.72]
Locke III	7	35	9	77	4.1%	1.89 [0.64, 5.57]
Siah	7	62	33	235	5.6%	0.78 [0.33, 1.86]
Zhao	106	147	2204	3001	12.8%	0.93 [0.65, 1.35]
**Subtotal (95% CI)**		**373**		**8434**	**38.7%**	**0.93 [0.72, 1.21]**
Total events	157		3547			
Heterogeneity: Tau² = 0.00; Chi² = 3.63, df = 4 (P = 0.46); I² = 0%						
Test for overall effect: Z = 0.52 (P = 0.60)						
**1.3.2 Hospital-based**						
Caballero-Plasencia	27	50	29	50	6.3%	0.85 [0.39, 1.87]
Chung	7	28	3	19	2.4%	1.78 [0.40, 7.97]
Corsetti	14	144	21	165	7.2%	0.74 [0.36, 1.51]
Nam	106	258	1224	2511	14.9%	0.73 [0.57, 0.95]
Salem	10	40	12	40	4.7%	0.78 [0.29, 2.08]
Sýkora	0	5	4	56	0.6%	1.06 [0.05, 22.42]
Yakoob	116	170	88	160	11.2%	1.76 [1.12, 2.75]
Yang	180	335	140	335	14.0%	1.62 [1.19, 2.20]
**Subtotal (95% CI)**		**1030**		**3336**	**61.3%**	**1.09 [0.74, 1.58]**
Total events	460		1521			
Heterogeneity: Tau² = 0.15; Chi² = 22.04, df = 7 (P = 0.002); I² = 68%						
Test for overall effect: Z = 0.43 (P = 0.67)						
**Total (95% CI)**		**1403**		**11770**	**100.0%**	**1.03 [0.80, 1.31]**
Total events	617		5068			
Heterogeneity: Tau² = 0.09; Chi² = 26.45, df = 12 (P = 0.009); I² = 55%						
Test for overall effect: Z = 0.20 (P = 0.84)						
Test for subgroup differences: Chi² = 0.42, df = 1 (P = 0.52), I² = 0%						

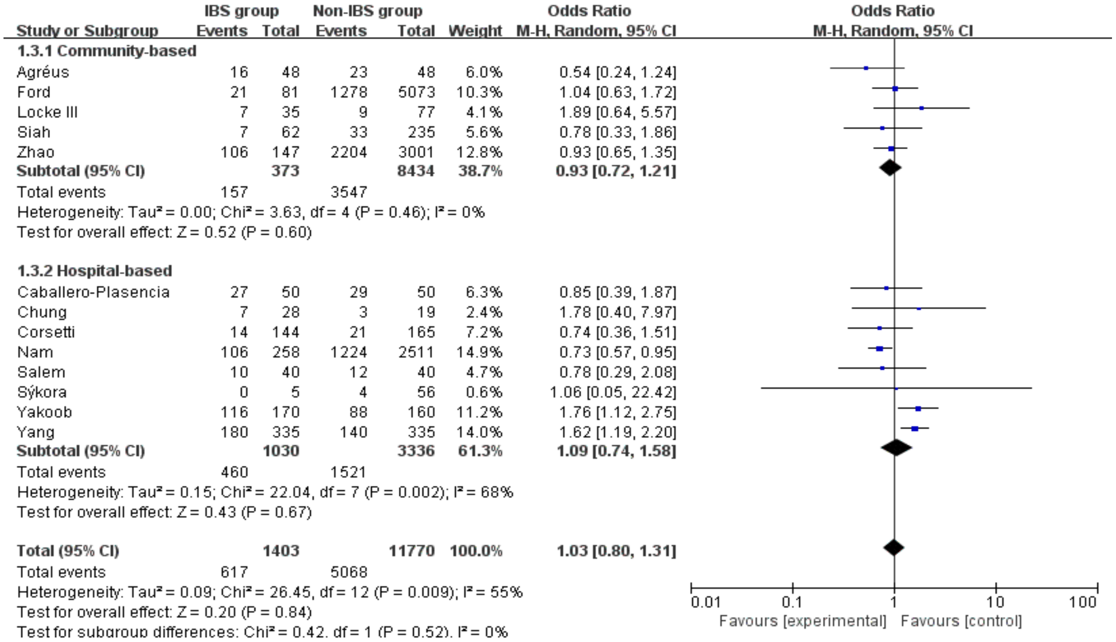

5.The forest plot of subgroup analysis stratified by study design

	IBS group		Non-IBS group			Odds Ratio
**Study or Subgroup**	**Events**	**Total**	**Events**	**Total**	**Weight**	**M-H, Random, 95% CI**
**1.10.1 Cross-sectional study**						
Ford	21	81	1278	5073	10.3%	1.04 [0.63, 1.72]
Locke III	7	35	9	77	4.1%	1.89 [0.64, 5.57]
Nam	106	258	1224	2511	14.9%	0.73 [0.57, 0.95]
Siah	7	62	33	235	5.6%	0.78 [0.33, 1.86]
Zhao	106	147	2204	3001	12.8%	0.93 [0.65, 1.35]
**Subtotal (95% CI)**		**583**		**10897**	**47.6%**	**0.85 [0.70, 1.04]**
Total events	247		4748			
Heterogeneity: Tau² = 0.00; Chi² = 4.25, df = 4 (P = 0.37); I² = 6%						
Test for overall effect: Z = 1.53 (P = 0.12)						
**1.10.2 Case-control study**						
Agréus	16	48	23	48	6.0%	0.54 [0.24, 1.24]
Caballero-Plasencia	27	50	29	50	6.3%	0.85 [0.39, 1.87]
Chung	7	28	3	19	2.4%	1.78 [0.40, 7.97]
Corsetti	14	144	21	165	7.2%	0.74 [0.36, 1.51]
Salem	10	40	12	40	4.7%	0.78 [0.29, 2.08]
Sýkora	0	5	4	56	0.6%	1.06 [0.05, 22.42]
Yakoob	116	170	88	160	11.2%	1.76 [1.12, 2.75]
Yang	180	335	140	335	14.0%	1.62 [1.19, 2.20]
**Subtotal (95% CI)**		**820**		**873**	**52.4%**	**1.13 [0.80, 1.60]**
Total events	370		320			
Heterogeneity: Tau² = 0.10; Chi² = 12.70, df = 7 (P = 0.08); I² = 45%						
Test for overall effect: Z = 0.67 (P = 0.50)						
**Total (95% CI)**		**1403**		**11770**	**100.0%**	**1.03 [0.80, 1.31]**
Total events	617		5068			
Heterogeneity: Tau² = 0.09; Chi² = 26.45, df = 12 (P = 0.009); I² = 55%						
Test for overall effect: Z = 0.20 (P = 0.84)						
Test for subgroup differences: Chi² = 1.82, df = 1 (P = 0.18), I² = 45.1%						

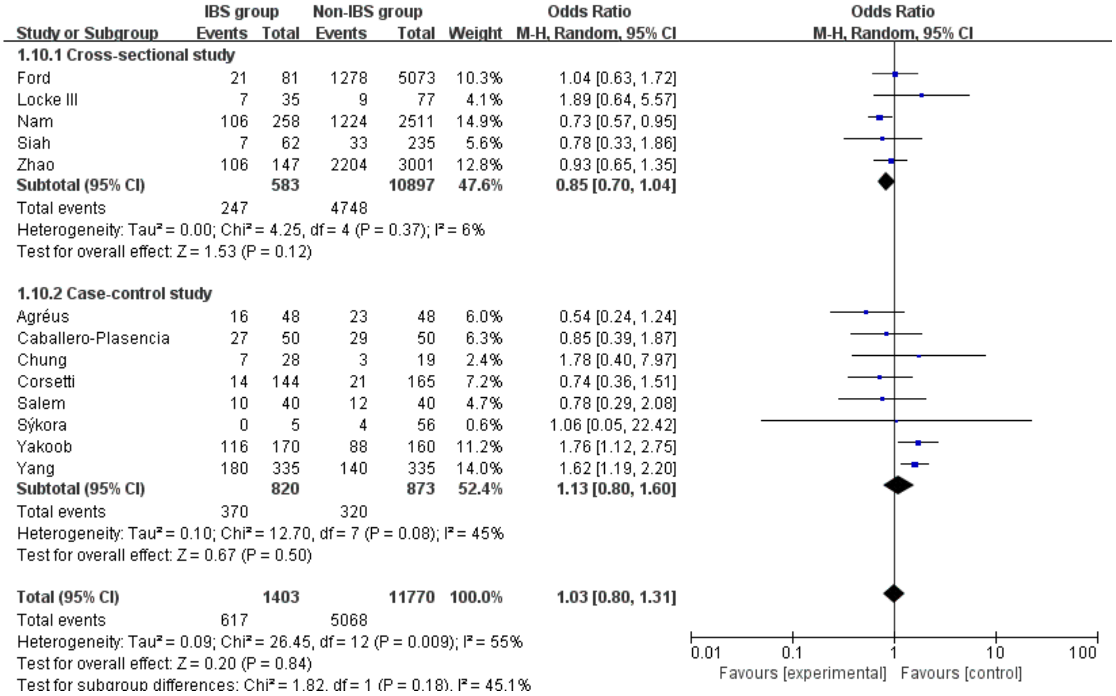

Supplement: Supplementary file 1 [file medicina-58-01035-s001.zip › Supplementary Material S4.pdf]
