# Supplementary figures and images for: Helicobacterpylori Infection—A Risk Factor for Irritable Bowel Syndrome? An Updated Systematic Review and Meta-Analysis
Source: Medicina (Kaunas). 2022 Aug 2;58(8):1035. doi: 10.3390/medicina58081035 (PMC9413972; doi:10.3390/medicina58081035)

## Supplementary Material S5. The figures of Egger's test and Begg's test

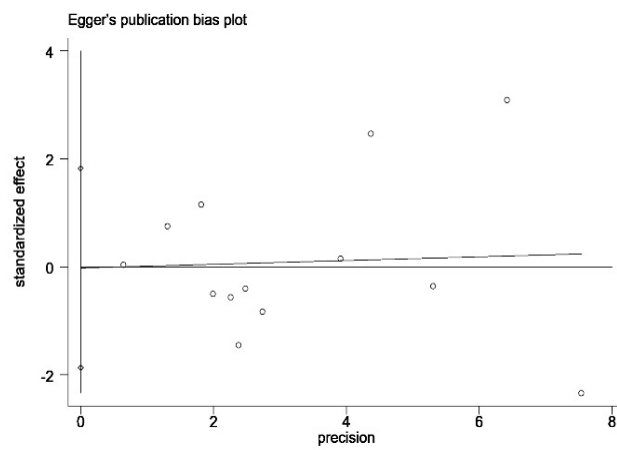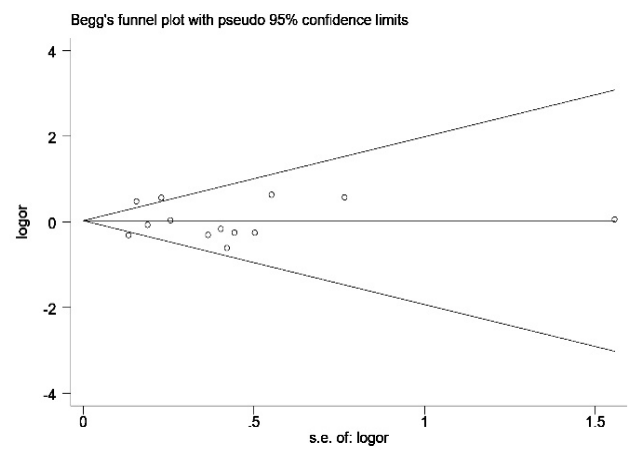

Supplement: Supplementary file 1 [file medicina-58-01035-s001.zip › Supplementary Material S5.pdf]
